# Assessing children’s competence to consent in research by a standardized tool: a validity study

**DOI:** 10.1186/1471-2431-12-156

**Published:** 2012-09-25

**Authors:** Irma M Hein, Pieter W Troost, Robert Lindeboom, Martine C de Vries, C Michel Zwaan, Ramón J L Lindauer

**Affiliations:** 1 Department of Child and Adolescent Psychiatry and de Bascule, Academic Medical Center Amsterdam, Meibergdreef 5, Amsterdam, 1105, AZ, The Netherlands; 2 Division of Clinical Methods and Public Health, Master Evidence Based Practice, Academic Medical Center Amsterdam, Meibergdreef 9, Amsterdam, 1105, AZ, The Netherlands; 3 Department of Medical Ethics and Health Law, Leiden University Medical Center, PO Box 9600, Leiden, 2300, RC, The Netherlands; 4 Department of Pediatric Oncology and Hematology, Erasmus Medical Center Rotterdam, Dr Molewaterplein 60, Rotterdam, 3015, GJ, The Netherlands

**Keywords:** Competence, Consent, Assessment, Tool, Drug trial, Informed consent, Decision making, Research, Child, Adolescent

## Abstract

**Background:**

Currently over 50% of drugs prescribed to children have not been evaluated properly for use in their age group. One key reason why children have been excluded from clinical trials is that they are not considered able to exercise meaningful autonomy over the decision to participate. Dutch law states that competence to consent can be presumed present at the age of 12 and above; however, in pediatric practice children’s competence is not that clearly presented and the transition from assent to active consent is gradual. A gold standard for competence assessment in children does not exist. In this article we describe a study protocol on the development of a standardized tool for assessing competence to consent in research in children and adolescents.

**Methods/design:**

In this study we modified the MacCAT-CR, the best evaluated competence assessment tool for adults, for use in children and adolescents. We will administer the tool prospectively to a cohort of pediatric patients from 6 to18 years during the selection stages of ongoing clinical trials. The outcomes of the MacCAT-CR interviews will be compared to a reference standard, established by the judgments of clinical investigators, and an expert panel consisting of child psychiatrists, child psychologists and medical ethicists. The reliability, criterion-related validity and reproducibility of the tool will be determined. As MacCAT-CR is a multi-item scale consisting of 13 items, power was justified at 130–190 subjects, providing a minimum of 10–15 observations per item. MacCAT-CR outcomes will be correlated with age, life experience, IQ, ethnicity, socio-economic status and competence judgment of the parent(s). It is anticipated that 160 participants will be recruited over 2 years to complete enrollment.

**Discussion:**

A validity study on an assessment tool of competence to consent is strongly needed in research practice, particularly in the child and adolescent population. In this study we will establish a reference standard of children’s competence to consent, combined with validation of an assessment instrument. Results can facilitate responsible involvement of children in clinical trials by further development of guidelines, health-care policies and legal policies.

## Background

### Introduction

Currently over 50% of drugs prescribed to children have not been properly evaluated for safety and efficacy in their age group. One key reason why children have been excluded from clinical trials is that they are not considered capable of understanding research information. This means that they are not considered able to exercise meaningful autonomy over the decision on trial participation. By Dutch law, competence to consent in children is presumed to be present at the age of 12 and above. In pediatric clinical practice, though, children’s competence is not that clearly presented. Children may express an increasing degree of competence over time as their abilities are developing, so there is a gradual transition from assent to the more active consent.

If we knew which children were competent to consent and which were not, it would be possible to involve them in the decision-making process about clinical trial participation in a conforming way. Non-competent children would no longer have to be burdened by the full informed-consent procedure, while competent children would be more actively engaged in that procedure, with extra weight given to their opinions. This would facilitate the implementation of clinical trials in children and adolescents and still protect the vulnerable subjects.

Previous studies show that children’s competence has never been systematically examined in a standardized manner [1]. The aim of this study is to develop a standardized competence assessment tool for children and to investigate the correlation between competence and age, IQ and patient characteristics. Once an objective tool for competence assessment becomes available, it can be implemented in inclusion stages of clinical trials in children.

This prospective observational study seeks to examine whether children’s competence to consent to research can be assessed in a reliable and valid way by means of a clinical tool.

### Background

#### Nature of competence

Individuals are competent if they are able to make decisions based on understanding and on rational reasons [2]. Competent decisions represent informed, free, self-determined choices and should be respected. This applies to informed consent as well as to informed refusal. Competence is task- and context-specific, which means that assessment of competence should be regarded as a specific judgment at a specific moment of the ability of the patient to fulfill the concrete task that he is facing [2]. Legal competence in health care requires being able to communicate a choice, understanding information one is given, appreciation of one’s circumstances and mental capacities to reason and deliberate [3,4].

#### Legislation

In current pediatric practice, parents and children undergo an informed consent procedure. According to the Dutch Medical Treatment Contracts Act (WGBO), which applies to treatment situations, parents decide for their children younger than 12 years of age, who are considered by definition incompetent to act for themselves. For these children, no actual assessment of competence is necessary. For children aged 12 to 15, informed consent is required both from children and parents, provided the children are judged competent to decide. From 16 years of age, children are deemed adult in medical decision making. In the Dutch Medical Research Involving Human Subjects Act (*Wet Medisch-wetenschappelijk Onderzoek met Mensen*, WMO), the same arrangements apply but age limits are set at 12 and 18 years of age. Children are deemed competent if they appear to understand information designed for their level of comprehension to an extent appropriate to the nature and scope of the decision. Internationally, the statutory age limits differ for clinical research: the lower age limit varies form 7 to 15, the upper age limit is set at 17 or 18 years [5].

In the Netherlands, the Doek Committee was installed to advise the State Secretary of Health, Well-being and Sports and the Minister of Justice [6] on the desirability of adapting the current regulations for medical scientific research with minors that does not benefit them directly. They concluded that the protection criteria in the Medical Research Involving Human Subjects Act give children little room to make decisions based on their own ideas and values. Children under 12 who wish to refuse participation in clinical research can formally only make that known through resistance. Children older than 12 may withhold permission, but if they do decide to participate, that can formally be nullified by their parents’ refusal. This conflicts with the prevailing view that children have the right to make decisions consistent with their value systems and life view. Children may be seen as moral agents, possessing autonomy [7]. The Doek Committee [6] therefore recommends that the will (the assent or refusal) of a younger child be taken into account to the degree that the child can understand the issues. The Task-force in Europe for Drug Development for the Young [8] issued recommendations to be implemented Europe-wide, stating, amongst other things, that minors should be involved in the informed-consent process in proportion to age and degree of maturity [65]. By assessing competence to consent in children and adolescents more objectively than is now the case, greater justice could be done to the ideal of respect for the developing autonomy of children in decision making, in accordance with national and international recommendations.

#### Competence assessment in current pediatric practice

At present, clinical investigators are able to make only intuitive assessments of children’s competence, because no standardized method is available to test it more objectively. Conceivably, the age standards prescribed by law may have too much influence on that intuitive decision by the assessor. Research evidence indicates that children under 12 may also be capable of making well-considered decisions [9,10] and that children as young as 9 can understand the issues involved in clinical trials [10]. This suggests a mismatch between children’s developmental level of decision-making maturity and the age limits set by legislation. The assessment of competence is subject to two pitfalls: complex research decisions may be imposed on children who are unable to make them, while capable children who want to take part may be inadvertently excluded [9]. It is recognized that age is, at best, a proxy for developmental capacity, and that experience, maturity and psychological state are key determining factors. This means that children’s competency to make important decisions ought to be assessed more individually than is presently the case.

A recent study has shown that doctors and researchers tend to judge a child as competent if the child’s decision conforms to their own ideas of the child’s best interest [11,12]. That means that competence is gauged by the content of the decision rather than by the *process of reasoning* in deciding about participation. It is therefore vital for clinical researchers to get access to a standardized, objective method to aid them in the informed-consent procedure, and thus improve the rigor of their judgment of children’s competence to consent.

#### Assessment of competence by MacCAT-CR

Until recently, literature on children’s competence assessment was limited. Two reviews [1,10] summarize the empirical literature on children’s competence to consent in research and treatment settings. A variety of different definitions and measures emerge from studies on children’s competence. Two quantitative instruments, namely the Competency Questionnaire – Child Psychiatric (CQ-ChP) [13] and the Hopkins Competency test (HCAT) [14], were examined, and some other authors (e.g. [15]) used semi-structured interviews. Only Weithorn’s interview [15] examined all four sub-domains of competence (see below).

Empirical studies on adults have resulted in a variety of operational translations of the concept of competence into assessment instruments. Dunn [16] assessed 23 existing instruments in terms of format, content, features of administration and psychometric properties and concluded that the MacArthur Competence Assessment Tools for Clinical Research (MacCAT-CR) and for Treatment (MacCAT-T) receive the most empirical support. These instruments have been tested in particular in samples of people with dementia, mental disabilities, schizophrenia and other psychiatric disorders. There are initial indications of validity [17] and a high degree of reliability [18].

The MacCAT-CR is a semi-structured interview format that helps clinical investigators to assess research candidates’ competence to give informed consent to participation in trials. It measures the four aspects of decision-making capability that reflect the standards for competence to consent in most jurisdictions: (1) *understanding* the disclosed information about the nature of the research and its procedures; (2) *reasoning* in the process of deciding about participation, with a focus on abilities to compare alternatives in the light of their consequences; (3) *appreciation* of the effects of research participation (or failure to participate) on their own situation; and (4) *expressing a choice* about participation [19]. Whilst an assessment of these abilities is essential, supplemental information may be needed about a candidate’s diagnoses, mental status and medical and social circumstances [19].

The MacCAT-CR provides a format for disclosing selected information on the research project at hand. A standard set of questions then assesses candidates’ abilities to understand the information, reason about it, appreciate its consequences and express a choice. The interview samples their abilities using representative content, rather than testing them on the full content of a typical informed-consent disclosure. The MacCAT-CR is based on the structure of the MacArthur Competence Assessment Tool for Treatment [17]. In the MacCAT-CR, the number and focus of questions in each section have been altered to suit a research context. Unlike the MacCAT-T, the MacCAT-CR questionnaire does not have to be individualized for each candidate. This facilitates both the research and the routine use of the interview.

The MacCAT-CR involves two steps: the interview itself (approximately 15, maximal 20 minutes) and the rating. The rating criteria provide a way for the assessing clinician to note opinions on the adequacy or inadequacy of each item response. The MacCAT-CR provides a summary rating for each of the four capacities assessed: 0 to 6 for understanding, 0 to 4 for appreciation, 0 to 8 for reasoning and 0 to 2 for expressing a choice [19]. A serious deficit in any of these abilities may translate to a clinical opinion of incompetence. The scale does not provide ‘cut-off scores’ that represent competence or incompetence, nor is there an overall MacCAT-CR total score [19]. The ratings provide the assessing clinician with a structured overview of the capacities needed for competent decision making.

In 2008, a Dutch translation of the MacCAT-T was made available by van Eyk [20]. Comments on the use of the MacCAT-T in the Dutch clinical setting were positive: it was judged as practicable and a valuable addition to current competence assessment practice [21]. Before administration, information on the content of the treatment needs to be thoroughly considered by the treating physician, which was viewed as a major advantage. The MacCAT-T aided the physician to structure the information required for the decision making of the individual patient [21].

In children, research on the MacCAT scales has been limited to two small studies. Koelch [22] used an adapted MacCAT-CR to study the decision-making process in 19 children aged 7 to 15 with psychiatric diagnoses; Tan [23] used the MacCAT-T to study thinking processes in 10 adolescents aged 13 to 21 with anorexia nervosa. Both studies confirmed the feasibility of using the MacCAT scales for children, but neither tested their validity and reliability More rigorous research is needed on the applicability of the MacCAT instruments for children.

#### Child-specific factors in competence judgment

1.Developmental aspects of competence

In children, decision-making abilities develop over time, as their cognitive, social and emotional abilities advance. Elementary school children develop the capacity for logical, systematic thinking using multiple pieces of information and the ability to perceive underlying reality despite superficial appearance. Cognitive advances, social relationships and emotional development work together to promote moral development in middle childhood and children become increasingly able to consider other people’s feelings [24]. But still, they face cognitive limitations: they lack the broad base of knowledge that adults possess. They still sometimes have trouble combining their cognitive skills into a larger problem-solving system. They cannot reason maturely about abstract and hypothetical problems [25]. In adolescence, the brain undergoes substantial change with an increase in efficiency of brain functioning. New cognitive skills such as hypothetico-deductive reasoning – the ability to think of hypothetical solutions and to formulate a systematic plan for deducing which of these solutions is correct – are acquired [24]. Social cognitive changes lead to increased maturity in reasoning about moral issues [25], giving space to altruism. Even with these advances, compared to adults certain cognitive limitations remain, mostly involving inconsistent application of recently acquired cognitive abilities [25].

The ability to balance risks and benefits depends on life experience as well as cognitive abilities. Children’s personal experiences of illness and their responses to it can provide them with greater insight and understanding than children of comparable age who lack this experience [26]. Over time, therapeutic relationships with children evolve and children grow and develop, and their response to the experience of illness alters. Competence assessment should respond to those changing circumstances.

2.Provision of information

Competence can be enhanced by improvement of information provision. Children and adolescents do not have the same comprehension level as adults. Their abilities to read and write and their working memory have not reached optimal growth yet. This implies that the information supply to children should be tailored to their developmental stage. Techniques for communication include both verbal and non-verbal forms of information supply, and breaking up the information into smaller pieces [26]. Information for children needs to be clearly worded, using simple language, and must connect to the perception of the child. Innovative techniques can contribute to conveying information [26]. Children sometimes need more time [26].

It has been found in current practice that communication with parents and children is often flawed [27-29] and even that children are incompletely informed [11]. As knowledge is a basic requirement for valid consent [30], assessment of competence needs to attend to the information process. Competence assessment taking the real-life situation as a starting point, containing the necessary information appropriate to the child’s level of development, provides the best basis.

3.Systemic influences

Growing-up children are, to a greater extent than adults, dependent on other people that surround them, especially their carers or parents. An exploration of the systemic influences is indispensable. Children may be particularly susceptible to the influence of parents and health-care professionals due to their need for approval or fear of negative consequences from authority figures [1,31]. Tates [32,33] states that the communication pattern in medical meetings is dominated by adults, and that physicians’ communication is parent-related. This illustrates the dependence of children on their parents and physicians for the information supply and level of involvement. Peer relations play an important role in development in middle childhood and adolescence. In interaction with friends, school-age children adhere very closely to peer group norms. In early adolescence, peer influence increases, and then declines [25]. Assessment of competence needs to pay attention to the influences of important others on the child’s decision-making process.

#### Modifications in MacCAT-CR for Children and Adolescents

The original MacCAT-CR by Appelbaum and Grisso [19] was translated into Dutch by a certified professional translator. In the next step the language was adapted to a simple level to be understood by children of elementary school age. We followed the guidelines of BureauTaal [34] to adjust the text to groups with low language skills. An expert on Dutch language and communication, who is also a teacher at the University of Primary Teachers, reviewed the text. Three child psychiatrists and three pediatricians carried out a final review of the text. In our study, the delivery of the interview has been customized to reflect the details of the particular trial in which candidates are asked to participate.

Ditters [21] says in his research that in Dutch clinical practice some wordings of the MacCAT-T interview and scoring manual are not familiar. Some of his interviewers showed difficulties understanding the conceptual framework, and they were uncertain as to when to probe for the right answer. We tackled this problem by applying simple language for the interviewers’ instructions and scoring manual as well. We added directions on when and how to probe, providing sample sentences. We aim to make the instrument available to different disciplines. Similarly, the patient information form and the informed-consent form for subjects in this study have been adapted to simple understandable language.

Additional to the worded information, Apppelbaum [19] recommends that subjects be given a card containing the disclosed information for each section and asked to read along as the disclosure is read to them. In children, reading performance, if present, might not have reached adult level. Equally, children might have problems dividing their attention between a written text and a spoken text. We thought it appropriate not to use text cards, and to provide cards with pictures disclosing information about medical procedures unfamiliar to the patient (e.g. an electroencephalogram) instead of written information.

The Dutch version of the modified MacCAT-CR for Children and Adolescents was translated back into English by another certified professional translator. One of the original authors of the MacCAT-CR, Appelbaum, provided comments on this version, which were processed. A special remark needs to be made on a modification to questioning the child’s understanding of the consequences of participating in the trial or not. We added the questions: “What do you think your parents will think about it if you take part or don’t take part? And what do you think your friends will think?” Scoring the answers, we note whether the child can mention consequences for daily life or social relations. With this approach we give more attention to the influence of social relationships than in the adult version of MacCAT-CR.

## Methods/design

This study is a prospective cohort study comparing competence judgment by using observational techniques to outcomes of the MacCAT-CR for Children and Adolescents, while at the same time assessing competency-related patient data.

### Methods/design

The validity and reliability of the translated and modified MacCAT-CR for Children and Adolescents will be assessed in a sample of children who are candidates to participate in ongoing medical trials. A reference standard for competence will be established first. The usual informed-consent procedure will be performed by the clinical investigators, and they will record their own intuitive clinical judgment of a child’s consent competence. At this point, parents will also be asked to judge whether their child has understood and is able to make a well-considered decision. This informed consent procedure will be videotaped. The recordings will then be reviewed independently by two experts (child psychiatrists, child psychologists or medical ethicists), who will also record a judgment on the child’s consent competence. This will allow us to estimate the inter-examiner reproducibility of the informed-consent procedure.

To establish a reference standard for consent competency, any discordant decisions for a child’s consent competency of the two experts and the clinician will be examined. If there is any discrepancy between the three evaluators’ judgments, a consensus decision will be reached after discussion. The final decisions will form the reference standard for competence.

After the usual informed-consent procedure, within 48 hours the MacCAT-CR interview will be administered by the researchers, independent of the first clinical judgment; these interviews will also be videotaped. The interviewers consist of specially trained special education or psychology graduates. The interviews will be scored by the administrator and independently by two experts (a multidisciplinary team of child psychiatrists, psychologists and medical ethicists). A yes/no decision will be made following the guidelines in the MacCAT-CR manual.

Demographic patient data will be collected and the Wechsler Nonverbal Scale of Abilities short version will be administered within two months of the interviews.

### Objectives

The final objectives are (1) to assess the reproducibility of MacCAT-CR scores and yes/no judgments of competence to consent, and (2) to establish a reference standard to which MacCAT-CR scores can be compared to evaluate the criterion-related validity of the instrument (in the absence of a criterion test for competence to consent). The reference standard will also be used to (1) estimate optimal cut-off scores on the MacCAT-CR scale that minimizes false positive and false negative decisions, and (2) determine ages for informed consent and compare these to current statutory age limits. The agreement between the reference standard and the judgment of the parent(s) will also be examined.

### Subjects

One hundred and sixty pediatric patients aged 6 to 18 who are involved in the selection stages of ongoing medical trials will be recruited. These will be trials that involve heterogeneous groups of children in terms of age and diagnosis (including childhood oncology, lung disease and ophthalmology). The children will be selected consecutively in the order of recruitment for the trials. The lower age limit of 6 is justifiable as younger children cannot be expected in developmental terms to be capable of meaningfully answering the interview questions. Age distribution will be structured in a way that approximately 70% of the sample (± 1 z-score) will be aged 8 to 14, because the transition point in competence is expected to occur there. The purpose is to avoid overestimating reproducibility and criterion validity as a result of excessive contrast in the age distribution. Grounds for exclusion will be insufficient fluency in Dutch.

### Measures

The outcome measures of the MacCAT-CR will be a total score, domain scores, and a binary assessment (yes/no) of a child’s competence to consent. Clinical investigators and parents will also be asked to give their prior intuitive yes/no assessments of the child’s competence to decide.

The Wechsler Nonverbal Scale of Ability short version (WVN) will be used to assess children’s intelligence quotient. The WNV is a clinical instrument for examining cognitive capacities of children and adolescents aged 4 to 21. The WVN is suitable for the general population as well as for children with cultural, linguistic, educational or socio-economic varying backgrounds. The subtests do not invoke verbal capacities as instructions are made by pictograms. Different subtests are to be administered in children from 4 to 7 and from 8 to 21. The short version for the first age group consists of matrix reasoning and recognizing, and for the second age group matrix reasoning and spatial orientation [35]. For practical reasons the short version is chosen: the two subtests take 20 minutes together. The full version gives more rigorous outcomes and has psychometrical advantages, but the validity and reliability of the short version are good. In this study the subjects cannot be burdened by the long version due to possible pain or distress. The WNV is the only IQ test with a standardized short version; this is in contrast to the Wechsler Intelligence Scale for Children. The WNV is approved by the Dutch Committee on Tests and Testing Affairs (COTAN). The WNV will be administered by trained certified professionals (special education or psychology graduates) under supervision of a senior professional.

The highest level of education of the highest-educated parent will be noted as an indicator of socio-economic status. Duration of disease, number of trials previously participated in and ethnicity will be noted.

### Informed-consent procedure

Prior written consent to take part in this study will be requested from all child participants and their parents, separate from any consent required of them for the drug trials. The pediatric patients sampled for the drug trials will be informed together with their parents by the trained clinical investigator about the competence study at the same time as they are informed about the drug trial. They will also be free to choose participation in one of the two studies with no consequences for the other. The outcome of the MacCAT-CR interview will not affect the conduct of the drug trial being carried out by the investigator. The ethics committee of the Academic Medical Center Amsterdam in the Netherlands confirmed that the Medical Research Involving Human Subjects Act (WMO) does not apply to this study and the committee makes no objections to the implementation.

### Statistical analysis

Descriptive summaries of demographic and assessment outcome measures will be generated with respect to all subject characteristics.

Reliability: Statistical analysis will include exploration of internal consistency, by estimating Cronbach’s alpha for the items of each subscale and for the total scale. We will also calculate adjusted item-to-scale total correlations. Factor analysis and item response theory methods (Rasch analysis) will be used to further test scale unidimensionality. To optimize the MacCAT-CR scoring system and to determine necessary item weights, we will use a specific extension of the Rasch measurement model One Parameter Logistic Model (OPLM) [36]. Conditional maximum likelihood estimation methods will be used to obtain stable item parameters.

Criterion-related validity: The overall accuracy of the MacCAT-CR score in classifying competence against the reference standard will be quantified using receiver operator characteristic curve (ROC) analysis. The area under the ROC curve (AUC) will serve as the validity coefficient; this may range from 50% (chance determination) to 100% (perfect determination). The optimal MacCAT cut-off score and the accompanying sensitivity and specificity rates will be determined using Youden’s method, the cut-off score corresponding to the fewest false positive and false negative classifications. Against our current expectations, this may give an indication of a single score above which competence is more likely. The validity of the current statutory cut-off ages will be tested by the same method, but using age as the predictor of capacity to provide informed consent.

Inter-rater reproducibility: We will determine inter-rater reproducibility (1) for the clinical judgment on competence to consent by the investigator and the experts, (2) for the MacCAT-CR total and subscale scores, (3) for the yes/no outcome of the reference standard and the yes/no outcome of the MacCAT-CR, and (4) for the yes/no outcome of the reference standard and the yes/no decision of the parents. We will quantify reproducibility with intraclass correlation coefficients for total scores on the MacCAT-CR scales. Inter-rater reproducibility of the item scores will be quantified by calculating weighted Kappa coefficients. To quantify the reproducibility of the yes/no outcome for competence to consent, multi-rater (unweighted) Kappa or simple Cohen’s Kappa will be used in the case of pairwise comparisons. We have a special interest in children between 8 and 14 years, and we will compare the ICCs and Kappa values calculated for children in this age group separately.

Statistical power analysis: There is no general agreement about estimating suitable sample size for the psychometric (factor-analytic, Rasch analytic) evaluation of multi-item scales. Simulation studies for the related techniques of regression analysis indicate that a minimum of 10 to 15 observations per variable (item) are needed to obtain stable estimates. For the 13 items of the MacCAT-CR this would result in 130 to 190 observations. Judging from previous studies on MacCAT-CR and MacCAT-T in adults with compromised decisional capacities [18,37-39], our proposed sample size of N = 160 is justifiable. In view of the three raters involved in our assessment of inter-rater reproducibility, the intraclass correlation for the MacCAT-CR score can be estimated with ±5% accuracy around the expected level of 0.80 with 95% certainty. The Kappa for the yes/no capacity to consent decision can be estimated with ±11% accuracy and 95% certainty assuming 60% raw agreement between two raters and an expected Kappa of 0.70.

An ROC-AUC validity statistic of 0.80 (null hypothesis AUC 0.69) can be detected with 80% power assuming a 3:1 ratio of test positive (competent to consent) and test negative children. Sensitivity and specificity rates of the MacCAT-CR cut-offs obtained by Youden’s method can be estimated with ±7% accuracy, assuming 0.75 as the expected value. For the Rasch analysis, the MacCAT-CR outcomes from the raters will be combined. Data dependency will not be an issue in our use of conditional maximum likelihood estimation methods, because the method makes no assumptions about the distribution of data in the population or about ways in which the sample was selected.

## Discussion

### Limitations

Due to the lack of a gold standard for competence assessment, the reference standard will be established according to current best clinical practice by physicians that deal with competence assessment in pediatric practice. The expert opinion represents the clinical conception of competence in children. These judgments constitute the best possible starting point. It is possible, however, that competence judgments vary between evaluators. Previous research demonstrates that unaided competence judgments, even of clinicians, are not reliable. Kim [40] describes considerable variety in competence judgments between experienced psychiatrists in a population of geriatric patients asked to participate in a hypothetical trial. Inter-rater reliability with group Kappa statistics ranges from fair to moderate agreement (0.40 to 0.45) for the psychiatrists’ judgments. The authors recommend more effective training in the judgment of competence to consent to research, as well as a judgment method. In this study this recommendation has been adopted: the expert panel that is to review the videos will be instructed and provided with basic information on competence judgment.

Another limitation might be that the same version of the MacCAT-CR will be administered to the whole range of children aged 6 to 18. Undoubtedly cognitive abilities and language skills vary widely in this age group. A pilot study did reveal some minor problems in 6-year-old children who did not understand the disclosed information and needed frequent rehearsal. Some of the older children understood easily, and they were not bothered by the simple language level.

### Recommendations

A validity study on an assessment tool of competence to consent is badly needed in research practice, particularly in the child and adolescent population. As far as we know, this is the first large-scale empirical study worldwide trying to establish a reference standard for children’s competence to consent, combined with validation of an assessment instrument. Results could lead to further development of guidelines, health-care policies and legal policies.

## Abbreviations

COTAN: Commissie Testaangelegenheden Nederland, Dutch Committee on Tests and Testing Affairs; MacCAT-CR: MacArthur Competence Assessment Tool for Clinical Research; MacCAT-T: MacArthur Competence Assessment Tool for Treatment; WGBO: Wet op de Geneeskundige Behandelovereenkomst, Medical Treatment Contracts Act; WMO: Wet Medisch-wetenschappelijk Onderzoek met Mensen, Medical Research Involving Human Subjects Act; ZonMW: The Netherlands Organization for Health Research and Development.

## Competing interests

The authors declare that they have no competing interests.

## Authors’ contributions

IH conceived the study and drafted the manuscript. PT, MdV, RL, MZ and RJL participated in the design of the study and coordination and helped to draft the manuscript. RL performed the statistical analysis. All authors read and approved the final manuscript.

## Pre-publication history

The pre-publication history for this paper can be accessed here:

http://www.biomedcentral.com/1471-2431/12/156/prepub
